# Segmentation, Tracing, and Quantification of Microglial Cells from 3D Image Stacks

**DOI:** 10.1038/s41598-019-44917-6

**Published:** 2019-06-12

**Authors:** Mahmoud Abdolhoseini, Murielle G. Kluge, Frederick R. Walker, Sarah J. Johnson

**Affiliations:** 1 The University of Newcastle, School of Electrical Engineering and Computing, Callaghan, NSW 2308 Australia; 2 The University of Newcastle, School of Biomedical Sciences and Pharmacy, Callaghan, NSW 2308 Australia; 3 The Hunter Medical Research Institute, New Lambton, NSW 2305 Australia

**Keywords:** Image processing, Programming language, Software

## Abstract

Microglia play a central role in modulating synaptic structure and physiology, learning and memory processes. They exhibit morphological changes to perform these roles, therefore the morphological study of microglia can help to understand their functionality. Many promising methods are proposed to automatically segment the blood vessels or reconstruct the neuronal morphology. However, they often fail to accurately capture microglia organizations due to the striking structural differences. This requires a more sophisticated approach of reconstruction taking into account the varying nature of branch structures and soma sizes. To this end, we propose an automated method to reconstruct microglia, and quantify their features from 2D/3D image datasets. We first employ multilevel thresholding to segment soma volumes(3D)/areas(2D) and recognize foreground voxels/pixels. Seed points sampled from the foreground, are connected to form the skeleton of the branches via the tracing process. The reconstructed data is quantified and written in SWC standard file format. We have applied our method to 3D image datasets of microglia, then evaluated the results using ground truth data, and compared them to those achieved via the state-of-the-art methods. Our method outperforms the others both in accuracy and computational time.

## Introduction

Glial cells are a family of cells that perform highly specialized roles within the central nervous system of mammals. Microglia, astrocytes and oligodendrocytes are three major sub-types of this family. Microglia have recently been identified to play a central role in modulating synaptic structure, synaptic physiology as well as learning and memory processes^1–3^. Microglia are not in permanent physical contact with other cells in their micro-environment. Therefore they need to engage in significant structural change to perform any of their known activities^4^. They exhibit distinct morphological transition states when undertaking specific biological functions. Therefore the morphological study of microglia is an important tool in understanding their functionality.

Manual reconstruction and quantification of the cells are very time consuming and prone to error, especially when dealing with 3D image stacks. Despite many promising automated methods in segmentation of blood vessels^5^, and reconstruction of neuronal morphology^6–11^, our evaluation results reveal that these methods often fail to accurately capture microglia organizations due to the striking structural differences. Soma size and branch architecture vary considerably between neurons, microglia and astrocytes but most importantly the morphology among microglia cells is highly variable. Neurons resemble a relative stable and reliable tree structure with a primary process, the axon, and multiple dendrites representing the tree trunk and canopy, respectively. Microglial architecture is more defuse and complex with multiple primary and secondary branches not confined to a generalizable structure. Additionally microglia morphology is highly diverse and changes by increasing and decreasing branches. This requires a more sophisticated approach of reconstruction taking into account the varying nature of branch structures and soma sizes.

Microglial cells consist of processes (branches) that form a tree structure and a central soma which envelops the cell nucleus. The easiest way to segment the outline of the cells is to distinguish the foreground from the background by calculating a single threshold based on the image intensity histogram^12^. Using this approach, a local iterative thresholding has been proposed to reconstruct microglial morphology^13^. This method is fast and easy, however a single threshold is not enough to preserve the topology of cells. Depending on the threshold value, either many fine processes may be missed or many delicate gaps may be filled with noise or unwanted objects.

A 2D segmentation of microglial cells via denoising and thresholding has been introduced^14^. A multifractal analysis has been performed on the segmented results to extract some spectrum features. Then a classifier is trained based on these features to classify microglial activation states. This approach is only applicable to 2D images, and the tree topology of the microglial cell is not considered. Therefore special features of the cells, such as branch points and branch length, cannot be truly quantified.

Discriminative dictionaries are another tool to recognize special patterns in images^15^. An over-complete dictionary via label consistent K-SVD^16^ has been learned to detect a sparse set of points (called seed points) that mostly lie on the image foreground^17^. A tree is grown for each cell by starting from a root node at the centre of its soma (previously segmented via a separate nuclear label) and connecting the seed points using geodesic distances and minimum spanning tree^18^. This approach requires accurate models (ground truth images) to learn a dictionary for each specific data set and the performance of the dictionary highly depends on the accuracy of the models. This approach outperforms open-curve active contour^19^ in which the foreground seed points are detected using a gradient vector flow field^20^. However, it has been reported that 27.8% of the seed points output by the dictionary are in the background^17^, which indicates a high potential of false traces.

Minimal path techniques have been employed to trace tree structural morphologies^21–23^. The tracing algorithm is started with a source point (manually chosen) on the tree structure and will stop at some end points which are either detected by a Harris detector^24^ or chosen from image border pixels. Each path between the source point and an end point is traversed by fast marching method^25^. This approach only segments an image containing a single cell from its border pixels and is very time consuming (computational time for a single structure in a synthetic image, size 220 × 300 is 94.94 seconds^22^). It also requires a user to manually choose a source point.

In this paper we propose a novel approach to segment and trace the microglial cells, and quantify their features from 2D/3D image datasets. The segmentation of the soma, and background removal via multilevel thresholding is the first step of our algorithm. Then a tracing step is initiated with the centroid of the somas and connects the prioritized seed points extracted from the cell voxels to build a tree structure for each cell. The thickness of the traced skeleton is estimated as the next step. Finally, the output data is quantified to extract the cell features such as: number of primary branches and branch points, branch length, soma size, and cell size. The whole of the process is automated and results in an objective morphological analysis of microglia with high speed and accuracy. The reconstructed data is written in SWC file format, so that it can be visualized or quantified via many toolboxes designed to read this standard format. As the experimental results, we have applied our method to 3D image datasets of microglia and compared the results with those achieved via the state-of-the-art algorithms. The comparison shows our method outperforms the others in terms of accuracy (assessed via four different metrics), and computational time.

## Method

### Soma segmentation and background removal

The first step is to locate the somas and extract the cell structures by removing the background. Let’s assume a 3D microscopy image stack of microglia (all the steps are applicable to 2D images as well) is converted to a gray-scale image in which foreground intensity is lower than background (i.e. dark cells on a light background). A 3D binary array, *B* with the same size as the image, *I*, is defined as follows:

1 $${B}_{t}(x,y,z)=\{\begin{array}{ll}1 & I(x,y,z)\le t;\\ 0 & {\rm{otherwise}},\end{array}$$

where *t* is a threshold value from the image intensity range. Each set of connected ‘1’s in *B* makes a 3D object. The number of 3D objects in *B* that are larger than a minimum (a suitable size for this minimum is discussed later) at a threshold *t* is denoted by *n*_*t*_. We are interested in a *t* in which these objects form the soma volumes with an acceptable precision. This threshold value is called *soma threshold*, denoted by *t*_s_. In the following, we propose a process of counting objects in different threshold values to find *t*_s_.

Intuitively as *t* increases, *n*_*t*_ will increase until it reaches the maximum. After that, growing *t* will decrease *n*_*t*_ due to objects starting to merge together. Tracking the changes of *n* with a proper set of ascending thresholds can help us to find *t*_s_. This proper set can be obtained from the image intensity histogram using multi-level Otsu’s method^12^. Using this method, 20 thresholds are chosen to assure smooth changes in *n*. This is more valuable than an arbitrary range of equally spaced thresholds which may include thresholds at levels of no value in demarcating image voxels.

**Figure 1:**
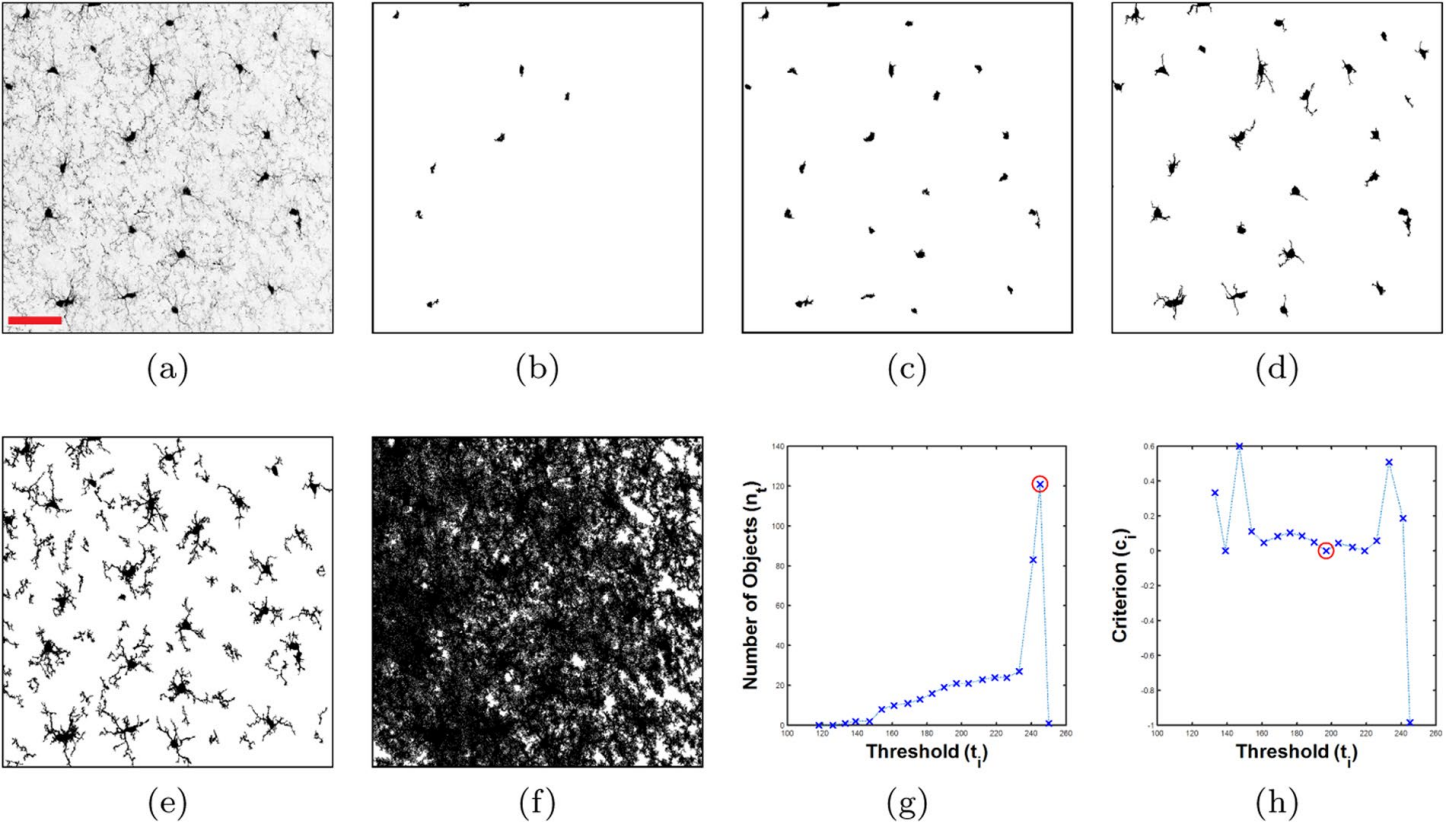
Soma segmentation and background removal using multiple thresholds. (**a**) Gray-scale MIP image, scale bar = 40 μm. (**b**) 2D MIP of ${B}_{{t}_{7}},\,{t}_{7}=161$. (**c**) 2D MIP of the soma array, ${B}_{{t}_{{\rm{s}}}},\,{t}_{{\rm{s}}}={t}_{12}=197$. (**d**) 2D MIP of ${B}_{{t}_{17}},\,{t}_{17}=233$. (**e**) 2D MIP of the cell voxel array, ${B}_{{t}_{{\rm{b}}}},\,{t}_{{\rm{b}}}={t}_{19}=245$. (**f**) 2D MIP of ${B}_{{t}_{20}},\,{t}_{20}=250$. (**g**) Number of objects (*n*_*t*_) vs. threshold (*t*_*i*_), circled cross represents the background level. (**h**) Criterion (*c*_*i*_) vs. threshold (*t*_*i*_), circled cross represents the soma level.

To consider changes in *n*, a normalized differential criterion, *c* is defined as follows:

2 $${c}_{i}=\frac{{n}_{{t}_{i+1}}-{n}_{{t}_{i}}}{{n}_{{t}_{i+1}}+{n}_{{t}_{i}}};\,\,{n}_{t} > 0,$$

in which *t*_*i*_ ∈ {*t*_1_, …, *t*_20_} is the set of 20 thresholds obtained by Otsu’s method, and sorted in ascending order. Assume for the first q thresholds, ${n}_{{t}_{i}}$ is growing, which means *c*_*i*_ is non-negative (i.e. *c*_*i*_ ≥ 0 where *i* = {1, …, q}). In this range, the *t*_s_ is empirically found to be where *n* has the minimum growing rate, which means *c* is minimum. If *c* reaches the minimum, *c*_min_, in more than one place (e.g. m places), i.e. *c*_min_ = min *c*_*i*_ where *i* = {*p*_1_, …, *p*_m_}, *t*_s_ is specified by the place where *c*_*i*+1_ − *c*_*i*_, *i* = {*p*_1_, …, *p*_m_} becomes minimum (Fig. 1(h)). The advantage of this approach is that it does not require the soma to be separately stained, however if this information is available, it can be used instead to locate the somas.

Also a threshold in which *n* reaches the absolute maximum can be considered as the *background threshold* denoted by *t*_b_. Because after *t*_b_ objects start merging together and forming one large solid which covers all the image array (Fig. 1(f)). Therefore voxel intensities larger than *t*_b_ have no value in demarcation of the foreground and will not participate in the cell tracing process. One way to find *t*_b_ is to calculate *n* for all the intensities and then find the absolute maximum. This is a slow process specially when the size of the image array is large. Another way of finding *t*_b_ is to use the values of *n* that have been already calculated for the threshold set, {*t*_1_, …, *t*_20_}. Assume *t*_max_ is a member of this set for which ${n}_{{t}_{{\rm{\max }}}}$ becomes maximum, so *t*_max_ will be either equal or very close to *t*_b_. Therefore, instead of searching all the intensities to find *t*_b_, we only need to search a small neighbourhood around *t*_max_. This makes it much faster to locate *t*_b_ for which ${n}_{{t}_{{\rm{b}}}}$ is the absolute maximum (Fig. 1(g)).

To accelerate the process of finding *t*_s_ and *t*_b_, a 2D version of the image *I* is defined as follows:

3 $$M(x,y)=\mathop{{\rm{\min }}}\limits_{z}\,I(x,y,z),$$

where $\mathop{{\rm{\min }}}\limits_{z}$ is the minimum along *z* direction. *M* is defined as a maximum intensity projection (MIP) of a 3D image stack when the foreground intensity is lighter (has larger value) than the background. Here, the reverse is true and the foreground is darker, therefore in (3) the minimum is applied instead of the maximum. For the purpose of finding *t*_s_ and *t*_b_ only a small thickness portion of *I* is used to create *M*. Specially it is important to consider a portion with the width of one cell approximately to avoid overlapping of too many cells (we use a thickness of 30 μm).

Definition (3) allows to find *t*_s_ and *t*_b_ through the 2D array *M* instead of the 3D image array *I*. This simplifies the process of counting objects from 3D to 2D and accelerates it a lot. To avoid counting noise or small isolated branches, we empirically found out only objects greater than 16 μm^2^ needed to be counted in this process. Note, although *t*_s_ and *t*_b_ are found using the 2D MIP, *M*, the full 3D image is thresholded to obtain *B* as shown in (1).

Voxels with intensities between {*t*_s_, …, *t*_b_} provide us with all the valuable information needed to trace paths through the centrelines of the processes. This is the advantage of multiple thresholds which provide several binary arrays, i.e. ${B}_{{t}_{{\rm{s}}}}$, ${B}_{{t}_{{\rm{s}}+{\rm{1}}}}$, ${B}_{{t}_{{\rm{b}}}}$, that participate in reconstruction: ${B}_{{t}_{{\rm{s}}}}$ shows all the soma volumes (Fig. 1(c)), ${B}_{{t}_{{\rm{b}}}}$ illustrates all the cell voxels (Fig. 1(e)), and ${B}_{{t}_{i}},\,i\in \{{\rm{s}}+\mathrm{1,}\ldots ,{\rm{b}}-\mathrm{1\}}$ include all the branch details that are necessary for tracing.

### Creating prioritized seed points

Only a small portion of the cell voxels (called seed points) are needed for the tracing stage. To extract the seed points, we define another binary array, *L* for the chosen threshold levels as follows:

4 $${L}_{i}={B}_{{t}_{i}}-{B}_{{t}_{i-1}},\,i\in \{{\rm{s}}+1,\ldots ,{\rm{b}}\}.$$

In each *L*_*i*_, ‘1’s specify places where voxel intensities of the image are between *t*_*i*−1_ and *t*_*i*_. These voxels are sampled to create the seed points.

In general, the closer a voxel is to the centreline of the processes, the darker intensity it has. Therefore, the target is to sample the voxels with low intensities in each level to be assured of smooth tracing on the centreline of the processes. Hence, the actual intensities of the cell voxels are needed during the sampling. To this end, ${\hat{L}}_{i}$ is created by element-wise multiplication of *L*_*i*_ by *I*, which makes the elements of ${\hat{L}}_{i}$ be equal to the image elements where *L*_*i*_(*x*, *y*, *z*) = 1, and ‘0’ otherwise.

The seed points are created by first dividing ${\hat{L}}_{i}$ into small volumes. Then the minimum intensity (i.e. darkest) voxel of each volume that consists of nonzero elements, is selected as a seed point. The size of the small volume specifies the sampling rate, the smaller the volume the higher the sampling rate. The sampling rate must be high enough to preserve the delicate curvy shape of the branches (in our experience, one seed point per 5 μm^3^ cube is sufficient).

In addition to the seed points sampled from the $\hat{L}$ arrays, a set of seed points sampled from soma surfaces is needed to assure us of tracing all the primary branches (branches that are directly connected to the soma). The surface of each soma is extracted from ${B}_{{t}_{{\rm{s}}}}$ and sampled (using the same procedure mentioned above) to form the set of seed points that initializes the tracing algorithm.

### Tracing algorithm

The seed points are sampled from different levels of intensity, starting from the soma surface with the lowest intensity to the last significant level, *L*_b_, with the highest intensity. For each cell, the tracing process is started with the centroid of the soma, ‘*root point*’, and ended with the last level, *L*_b_. This helps to direct the path through the centreline of the processes and smoothly reconstruct the skeleton of the branches. Each cell is traced individually inside a defined boundary to build a tree-like structure. The default boundary is set to be large enough (a cube with 60 μm each side) to cover all the microglial cell structure.

A connected set is defined as a set that consists of the seed points already joined to the cell tree structure during the tracing process. A disconnected set is defined as a set of seed points that are not part of the tree yet but are sufficiently close to an element in the connected set. The connected set is initialized to be the root point and all the seed points from the soma surface connected to it. The disconnected set is initialized with seed points that exist within a small volume around each seed point of the soma surface. The small volume size specifies a desired distance within which the seed points are reachable. Depending on the sampling rate, this size must be large enough (a cube of 40 μm^3^ in our experiment) to assure all the seed points lying on the cell structures are reachable.

After initialization, a loop is designed to withdraw a seed point from the disconnected set and place it inside the connected set in each iteration. The chosen seed point is the one with a minimum geodesic distance to a member of the connected set. The geodesic distances are calculated between the members of the sets as follows. Assume ‘k’ is a member of the connected set for which we want to calculate the geodesic distances. ${B}_{{t}_{{\rm{b}}}}$ is cropped with the specified volume around ‘k’ to obtain an object which includes ‘k’. Then Euclidean distances are calculated from ‘k’ to members of the disconnected set which belongs to this object. Those which do not belong to this object will be considered as the seed points with infinite distances and discarded.

After joining a new point to the connected set, the disconnected set will be updated by the new point’s surrounding seed points within the specified volume. Then the same process will be repeated in the next iteration to specify a new connection. The tracing process will be continued until all the reachable seed points that belong to the first level, *L*_*s*+1_ are connected. Then, it steps up to the next level, *L*_*s*+2_ and connects all the reachable seed points in this level to the tree with the same procedure. After connecting all the reachable seed points in each level, it steps up to the next higher level until all the reachable seed points from all the levels are connected. The entire process is automated with zero user interaction required.

During the process of tracing centrelines, many small branches are added to the cell tree structures. A pruning stage is implemented after the tracing to remove branches shorter than a specified minimum. We set this minimum to 5 μm as microglial branches smaller than this length are difficult to resolve.

In 2D reconstruction, there might be some branches that start from a cell and go to another with the same intensity along the way due to the maximum intensity projection. In these cases, there must be another decision metric other than intensity to cut the branch at some point along its path. We found out the watershed algorithm^26–28^ is an appropriate tool to make such a decision. Note, this problem no longer exists in 3D reconstruction of the cells from 3D image stacks, since there is no projection and 3D branches have different intensity along the way. Therefore, the application of watershed is no longer recommended.

### Finding the thickness of the branches

Although the pruned short branches are not part of the skeleton, they in fact represent the thickness (or the radius of the cross section) of the main branches that form the skeleton. Therefore their locations and lengths are employed to estimate the branch radii.

After the pruning process, all the points (except the root point) which form the tree structure of a cell can be divided into two categories. (1) Points to which small branches were attached before pruning, where the radii are defined as equal to the length of these branches. (2) Points which did not hold any extra small branches, where the radii must be estimated using the radii at the neighbouring points.

For each traced cell skeleton, the radius estimation starts from the root point and spans the tree from the root to the tip of the branches. The root point’s children (points on the soma surface that are directly connected to the root point) are the starting points of the branches. If the radius is not specified at a starting point, the radius at its child location can be a good estimation, since branch thickness is generally a smoothly changing value along the length of a branch. Except the starting points, if the radius is not specified at any other point on the branch centreline, it is estimated by the average of the radii at its parent and child locations. After estimating the radii at all the points, they are stored in a radii vector.

### Quantification

Some features that help neuroscientist to gain good insight into the cell functionality are extracted and quantified using the output of the proposed method. These quantified features are: number of primary branches, number of branch points, branch length, soma size, and cell size.

Primary branches are the main branches that are directly connected to the soma. Secondary and tertiary branches divide from primary as it grows. The number of primary branches equals the number of the root point’s children which is an output in our method. A branch point is a place where the processes bifurcate. Therefore the number of bifurcations equals the number of points that have more than one child. Branch length is equal to the summation of all the segment lengths that form the cell tree structure.

Soma size is directly obtained from the ${B}_{{t}_{{\rm{s}}}}$ array that consists of the soma volumes (3D) or area (2D). Cell size is quantified by calculating the cell volume in 3D or cell area in 2D. The radii vector calculated in previous section, represents the radius of a branch cross section in 3D (or the length of a perpendicular line to the centreline in 2D) at each point that lies on the centreline of the branches. Therefore the volume, *v*, of a solid segment between each pair of points is calculated as follows:

5 $$v=\frac{\pi }{3}\times h\times ({r}_{1}^{2}+{r}_{1}{r}_{2}+{r}_{2}^{2}),$$

if it is 3D or the segment area, *a*:

6 $$a=h\times ({r}_{1}+{r}_{2}),$$

if it is 2D, where *r*_1_ and *r*_2_ are the radii at the beginning and end points of the segment respectively, which are obtained from the radii vector, and *h* is the length of the segment. The cell size is equal to the summation of all the segment volumes/areas (starting from root point’s children to the end points of the branches) calculated via (5) or (6), plus soma size.

### Output in SWC file format

The traced path output has a tree structure for each cell and is written in SWC file format. SWC is a standard ASCII format including all the information necessary for quantification and visualization of the cells. Each point on the traced path is assigned a row with seven fields in the SWC reconstruction file. These fields are: index number, tag number (which specifies different parts of a cell, e.g. ‘1’ is assigned to soma), three spatial coordinates, radius, and parent index^29^. There are some tools publicly available to read, visualize, and quantify this format, such as: Neuromantic^30^, SharkViewer^31^, Vaa3D^32^, and L-Measure^33^.

The SWC format is also employed to assess the quality of our reconstruction. One way to evaluate the performance of a method and compare it to other methods is using DIADEM metric^34^ to benchmark automated reconstructions against manual (ground truth). DIADEM is a multi-step scoring process designed for tree-like structures, which takes into account the connections between points, bifurcation coordinates, excess and missing branches. Depending on the dataset, DIADEM requires some parameters to be set before running the metric. For all of our experiments, we set these parameters as follows: xy-path-thresh = 1, z-path-thresh = 1, terminal-path-threshold = 20, terminal-threshold = 15, xy-threshold = 10, z-threshold = 8, and left the remainder as default.

Another way of performance evaluation is to measure spatial Euclidean distances between the nodes of two reconstructions (automated and ground truth). These measures are spatial distance (SD), substantial spatial distance (SSD), and the percentage of SSD nodes (SSD%)^32^. We calculate these measures via NDIST^35^ with the default value of 2 voxels for the substantial distance. Both metrics receive SWC files to perform their evaluation.

### Image acquisition

All experiments were carried out in 6–8 weeks old male Cx3CR1^GFP/+^ mice, expressing fluorescently labelled microglia. Animals were obtained from the Garvan Institute of Medical Research and Australian BioResources, Sydney. All experiments were approved by the University of Newcastle Animal Care and Ethics Committee, and conducted in accordance with the New South Wales Animals Research Act and the Australian Code of Practice for the use of animals for scientific purposes. High resolution confocal images of fixed brain sections were taken on a Leica TCS SP8 confocal microscope with a Leica HC PLC APO 40x/1.30 OIL objective. Image stacks of the entire 30 μm slices were taken at 0.2, 0.5 and 1.0 μm *z*-step sizes.

## Results

Two experiments are performed in this section: 1- reconstruction of 21 microglial cells and benchmarking the results against the ground truth images and comparing them to that of the state-of-the-art methods, 2- testing the ability of our method in reconstruction and quantification of large 3D image stack.

### Performance evaluation

We have applied our method to 21 microglial images including 3D image patches of different sizes from 201 × 201 × 26 to 401 × 401 × 108 voxels. 18 of these images were imaged in our own lab, and three of them are available here^17^. We have benchmarked the quality of our reconstructions against ground truth images (manually reconstructed images by experts via Neuromantic^30^) using 4 different metrics: SD, SSD, SSD%, and DIADEM to evaluate the efficiency of our method. The results have been compared to that of four state-of-the-art methods: Multiple Neuron Tracer (MNT)^17^, App2^10^, Simple Tracing (ST)^11^, and Ensemble Neuron Tracer (ENT)^9^ (last three methods are implemented as plug-ins inside Vaa3D framework, version 3.200^32^). The parameters in each application were tuned through an exhaustive search to get the best results as follows. Ensemble Neuron Tracer (ENT) and Simple Tracing (ST) do not require any parameter. As for APP2 the parameters were set as follows, length_thresh = 5, cnn_type = 2, and background_threshold was set to be automatically chosen since any changes result in invalid outputs. As for MNT the parameters were set as follows, cost_threshold = 1000, intensity_threshold = 0.005, and contrast_threshold = 0.0003. As for our method, the parameters are chosen based on the expected size of microglial cells as explained in the method section and would not normally need to be adjusted by the user. Note, the fluorescent images are RGB color images with the green channel containing the signal. Thus the green channel was specified prior to running the plugins in Vaa3D for each plugin that required a channel specification. Also note, soma centroids must be provided before running MNT which is a disadvantage of this method, and SWC files produced by ENT are slightly different from the standard definition and cannot be assessed by DIADEM metric.

**Figure 2:**
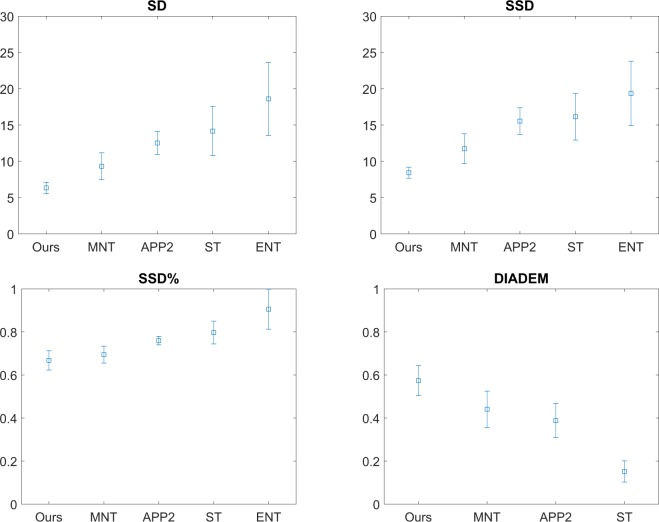
Mean and its 95% confidence interval plots of the evaluation results summarized in Table 1, achieved via our method, Multiple Neuron Tracer (MNT), App2, Simple Tracing (ST), and Ensemble Neuron Tracer (ENT).

Table 1 shows the means and standard errors of the results. They are also plotted with 95% confidence interval of the mean in Fig. 2. The comparison shows our method achieves the lower spacial distances (SD, SSD, and SSD%) to the ground truths, and the higher DIADEM scores. This means that the images reconstructed via our method are more similar to the ground truths than the results produced via the state-of-the-art, therefore our method outperforms them in terms of quality of reconstruction assessed via these metrics.

**Table 1:** Average and standard errors of evaluation results. MNT: Multiple Neuron Tracer^17^, App2^10^, ST: Simple Tracing^11^, ENT: Ensemble Neuron Tracer^9^.

		Ours	MNT	App2	ST	ENT
Mean	SD	6.34	9.32	12.53	14.16	18.60
	SSD	8.43	11.74	15.54	16.14	19.36
	SSD%	0.67	0.69	0.76	0.80	0.91
	DIADEM	0.57	0.44	0.39	0.15	NA
Std. error	SD	0.39	0.93	0.78	1.69	2.52
	SSD	0.37	1.02	0.91	1.61	2.22
	SSD%	0.02	0.02	0.01	0.03	0.05
	DIADEM	0.03	0.04	0.04	0.02	NA

**Figure 3:**
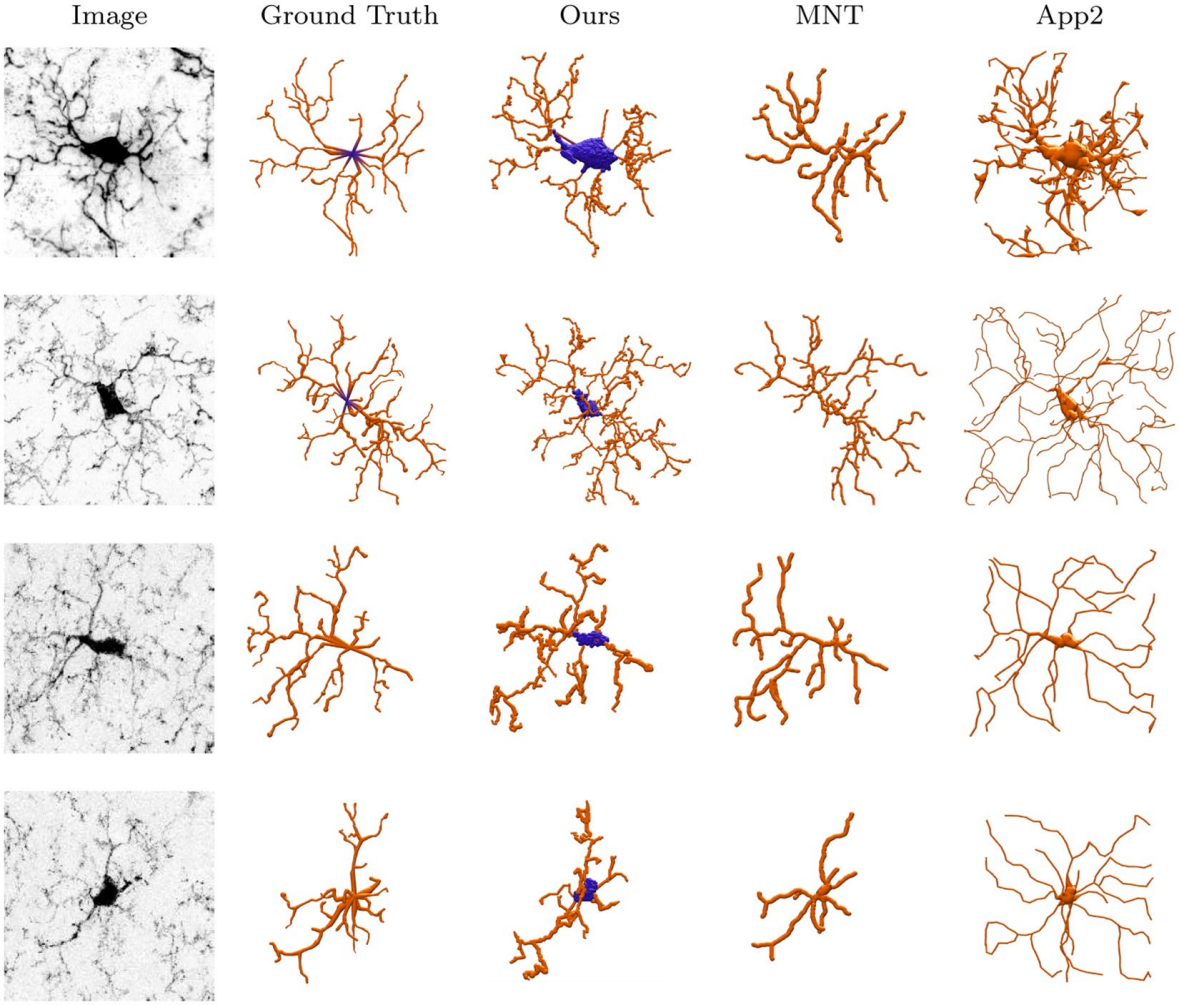
3D visualization of 4 microglial samples reconstructed via our method, MNT^17^, and App2^10^.

Four SWC files of the reconstructed cells are randomly selected to be visualized via SharkViewer^31^ (Fig. 3). It can be seen that our 3D reconstructions are the closest to the ground truths. Since soma volumes are automatically segmented in our method, they are also visualized in Fig. 3. Note, somas are not considered in the performance evaluation via the employed metrics.

It is hard to compare the computational time of these algorithms, since they are coded in different programming languages (ours in MATLAB, the other methods in C++) and optimized for different platforms. However, Table 2 summarize the computational time of these methods to give us a clue about their computational order. These times have been recorded while these methods are applied to the same image with a size of 201 × 201 × 26, running on a DELL core i7 laptop with 16 GB RAM. It shows that our method reconstructs the image faster than others.

**Table 2:** Computational time of different methods. System specifications: Windows 10 running on Dell, Intel core i7 with 16 GB memory. Image size: 201 × 201 × 26.

	Ours	MNT	App2	ST	ENT
Time (seconds)	1.19	8.46	4.56	1.23	34.07

### Reconstruction and quantification of a large size image

**Figure 4:**
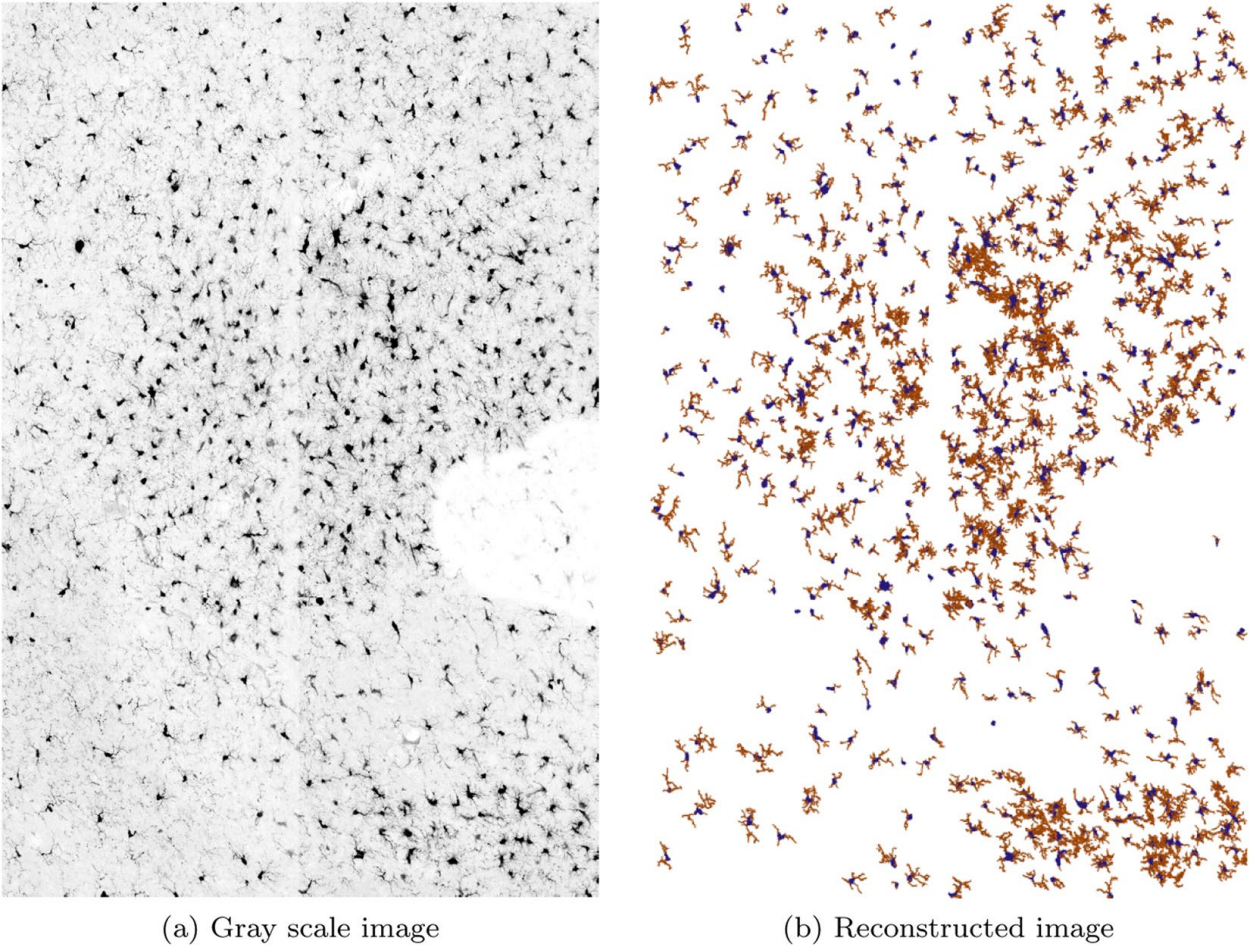
3D reconstruction of microglial cells from a fluorescent image stack. Dimensions: 3070 × 2047 × 21 voxel equals 1108 μm × 738 μm × 30 μm physical size. (**a**) Gray scale version of the maximum intensity projection of the original image, (**b**) 3D reconstruction.

We have applied our method to a large 3D fluorescent image stack (dimensions: 3070 × 2047 × 21 voxel equals 1108 μm × 738 μm × 30 μm physical size) of microglia including 532 cells. The 3D reconstruction is visualized in Fig. 4 via SharkViewer^31^, and the average quantitative results of the cell features are presented in Table 3. The computational time required to automatically reconstruct and quantify the whole of the 3D stack was 415 seconds. These results show that our method is successful in reconstruction of large 3D microglial images, and quantification of their features with high speed and accuracy.

**Table 3:** Quantification results. NPB: number of primary branches, NBP: number of branch points. The branch length is in μm, and the soma and cell sizes are in μm^3^.

	NPB	NBP	Branch length	Soma size	Cell size	Time (seconds)
Average per cell	5.12	11.31	230	124	520	0.78

## Discussion

We have presented a completely automated fast algorithm for reconstruction of microglial cells from 3D and 2D microscopy images which allows accurate quantification and visualization of the traced cells. Automatic soma segmentation and background removal are performed using multilevel thresholding as the first step of our algorithm. Different intensity levels output by the first step are employed to create the prioritized seed points. The tracing step is started with the centroid of the soma found in the segmentation step and ended with the background level found in the background removal step.

Prioritizing the seed points based on their intensities helps to smoothly direct the paths through the centreline of the processes. The definition of the geodesic distance via the cell voxel array, ${B}_{{{\rm{t}}}_{{\rm{b}}}}$, helps to avoid incorrect connection between the seed points during the tracing process. This process is designed to connect one disconnected seed point to the path in each iteration. This prevents a loop in the path and ensures a tree-like structure output for each cell which fulfils the topology of the microglial cell. The thickness of the traced centrelines are estimated through the length of small branches attached to them and used to derive area/volume measures. The reconstructed cells are written in SWC format and can be quantified and visualized to provide the morphological features of the cells which are crucial to gain insight into the cell functionality.

We evaluated the performance of our method and compared it with four state-of-the-art methods by applying them to 3D image stacks of microglia. The results were benchmarked against the ground truth images using four different metrics. The comparison has shown our method achieves the lower spacial distances to the ground truths and the higher DIADEM scores which means our method outperforms the others in terms of the reconstruction accuracy. We have also demonstrated the satisfying performance of our method in reconstruction of large 3D microglial images, and quantification of their features.

So far, the branch growing algorithms use intensity and local proximity information to decide how to trace the cells. On the other hand in the manual reconstruction, an expert uses information about the angle of the branch at the connection and the slight thickening at the connection point to determine where the branches should be broken, or might decide to ignore a gap and connect a broken branch due to biological features. As a future work, we suggest to consider these biological features of the microglia to create a smart tracing algorithm which incorporates this expert knowledge directly.
